# Transcriptome analysis of *Spodoptera frugiperda* Sf9 cells reveals putative apoptosis-related genes and a preliminary apoptosis mechanism induced by azadirachtin

**DOI:** 10.1038/s41598-017-12713-9

**Published:** 2017-10-16

**Authors:** Benshui Shu, Jingjing Zhang, Veeran Sethuraman, Gaofeng Cui, Xin Yi, Guohua Zhong

**Affiliations:** Key Laboratory of Crop Integrated Pest Management in South China, Ministry of Agriculture, Key Laboratory of Natural Pesticide and Chemical Biology, Ministry of Education, South China Agricultural University, Guangzhou, People’s Republic of China

## Abstract

As an important botanical pesticide, azadirachtin demonstrates broad insecticidal activity against many agricultural pests. The results of a previous study indicated the toxicity and apoptosis induction of azadirachtin in *Spodoptera frugiperda* Sf9 cells. However, the lack of genomic data has hindered a deeper investigation of apoptosis in Sf9 cells at a molecular level. In the present study, the complete transcriptome data for Sf9 cell line was accomplished using Illumina sequencing technology, and 97 putative apoptosis-related genes were identified through BLAST and KEGG orthologue annotations. Fragments of potential candidate apoptosis-related genes were cloned, and the mRNA expression patterns of ten identified genes regulated by azadirachtin were examined using qRT-PCR. Furthermore, Western blot analysis showed that six putative apoptosis-related proteins were upregulated after being treated with azadirachtin while the protein Bcl-2 were downregulated. These data suggested that both intrinsic and extrinsic apoptotic signal pathways comprising the identified potential apoptosis-related genes were potentially active in *S*. *frugiperda*. In addition, the preliminary results revealed that caspase-dependent or caspase-independent apoptotic pathways could function in azadirachtin-induced apoptosis in Sf9 cells.

## Introduction

Sf9 cells, derived from the immature ovaries of fall armyworm moth (*Spodoptera frugiperda*) pupae^1,2^, have become one of the most widely used insect cell lines for eukaryotic protein expression with the advantages of high protein expression and easy manipulation^3^. Interestingly, reflecting inerratic characters and easy culture^4^, Sf9 cells are also regarded as an ideal model organism for cytotoxicity screening and apoptosis research, and although apoptosis in Sf9 cells has been extensively studied, there is deficient research on its comprehensive genomic resource^5,6^. Next-generation sequencing (NGS) technology is a revolutionary change over traditional sequencing, which has been widely used for *de novo* genome sequencing, re-sequencing, small RNA sequencing and SNP discovery^7^. Therefore, the abundant sequence data generated using the NGS technique could be an abundant resource for deep and systematic studies in apoptosis.

Apoptosis is an autonomic-ordered cell death process that independently eliminates superfluous or unwanted cells to maintain the balance of homeostasis in multicellular organisms in response to internal and external stimuli, such as hormones, viruses, UV rays, botanical pesticides, etc.^8–10^. Among insect, apoptotic mechanisms of *Drosophila melanogaster* is more comprehensive. The occurrence of apoptosis process is mainly dependent on cysteine proteases called caspases. Seven caspases were identified in *D*. *melanogaster* and divided into two categories: initiator caspases (Dronc, Dredd and dream) and effector caspases (Drice, Dcp-1, decay and Damm)^11^. The first step of apoptosis process is the activation of initiator caspases and an octameric apoptosome assembled by Dark recruit and interact with Dronc, forms a single-ring apoptosome and the activated Dronc^12^. The activated Dronc then activates DrICE. The activated DrICE was response for cleavage of cellular components, leading cell to death by various morphological changes and biochemical events, like membrane blebbing, cell shrinkage, formation of apoptotic bodies and DNA fragmentation^13,14^. Many apoptotic factors involved in the process were revealed. The formation of the apoptosome and caspase activation in *Drosophila* seem doesn’t require the participation of cytochrome c^15^. DIAP1 could interacts with Dronc, Drice and Dcp-1 and block the activation of caspases by ubiquitination activity^16–18^. The IAP antagonists named as RHG (Reaper, Hid, Grim and Sickle) family proteins promote apoptosis by competitive binding to IAP with caspases^19^. At present, apoptosis study in Sf9 cells still stay in the level of physiological and biochemical changes and cloning and function analysis of a small amount apoptosis genes, and apoptosis mechanisms of *D*. *melanogaster* can’t fully reflect the apoptotic mechanism of Sf9 cells, comprehensive and in-depth study of apoptosis is indispensable.

Azadirachtin, a natural tetranortriterpenoid compound, has been demonstrated as one of the most promising plant compounds for pest control in organic agriculture^20,21^. Previous studies have confirmed that its strong antifeedant and growth-regulating activities and sterility effects could be the most important mechanisms underlying the actions of azadirachtin^22,23^. In recent years, the study of apoptosis induction in many insect cell lines, including Sf9, Sl-1 (*S*. *litura*), BTI-Tn-5B1–4 (*Trichoplusia ni*) and S2 (*Drosophila melanogaster*), has become another research hotspot of azadirachtin *in vitro* ^24–27^. Several apoptotic signalling pathways have been verified as activated in apoptosis induced by azadirachtin. For example, mitochondrial signalling is activated in apoptosis induced by azadirachtin in Sf9 cells with the evidence of physiological aspects and cytochrome *c* release^28^. The lysosomal signalling pathway plays a crucial role in apoptosis induced by azadirachtin, and cathepsin L exerts its function as a prop-apoptotic factor by engaging the release of cathepsin L into the cytosol and activating caspase-3^29^. The activation of Ca^2+^-CaM and EcR/Usp signalling pathways were confirmed in S2 cell apoptosis induced by azadirachtin^27^, and PI3K/AKT/TOR pathways were revealed to regulate the transformation of autophagy and apoptosis induced by azadirachtin in SL-1 cells^30^. Azadirachtin induced apoptosis in different cells through the activation of different signalling pathways, and whether these pathways also exist in Sf9 cells and are involved in the apoptosis induced by azadirachtin remains unclear.

To analyse the mechanism of apoptosis induced by azadirachtin in Sf9 cells, the present study investigated the transcriptome of Sf9 cell line using Illumina platform. A total of 87,860 unigenes were obtained, and 97 apoptosis-related genes were identified. RT-PCR was used to clone 15 candidate apoptosis-related genes, and the expression patterns of ten selected apoptosis-related genes were compared using qRT-PCR between azadirachtin-treated and untreated cells. Furthermore, the results of Western blotting verified roles for seven proteins in apoptosis induction by azadirachtin in Sf9 cells. These results not only enrich the transcriptome diversity of *S*. *frugiperda* and contribute to the identification and validation of apoptosis-related genes in Sf9 cells, but also reveal the preliminary apoptosis mechanism of azadirachtin.

## Results

### Transcriptome sequencing and sequence assembly

The transcriptome analysis of Sf9 cells in the present study contained approximately 48 million raw reads and approximately 47.5 million clean reads generated after removing reads containing adapters, reads containing poly-N and low-quality reads. The clean reads data has been submitted to the SRA database with the accession number of SRR5892097. Additionally, the error rate, Q20, Q30 and GC-content of the clean reads were 0.01, 98.07%, 95.14% and 45.81%, respectively. In total, 103,977 transcripts were assembled using Trinity, and 87,860 unigenes were generated after selecting the longest transcript of each gene as the unigene. The N50 length and mean length of total unigenes was 1182 and 672 bp, respectively, ranging from 201 to 29,609 bp. Unigenes ≥ 2000 bp accounted for 6.62% of the total unigenes (Figure S1).

### Functional annotation of the transcriptome

The results of the functional annotation of the unigenes in seven databases are shown in Table 1. The numbers of unigenes annotated in the NR, GO, PFAM and Swiss-Prot were 16,233 (18.47%), 13,458 (15.31%), 13,343(15.18%) and 10,836 (12.33%), respectively. In addition, only 22,722 unigenes (25.86%) were annotated in at least one database.

**Table 1:** Statistics of Gene annotation success rate.

	Number of Unigenes	Percentage (%)
Annotated in NR	16233	18.47
Annotated in NT	10198	11.6
Annotated in KO	6754	7.68
Annotated in SwissProt	10836	12.33
Annotated in PFAM	13343	15.18
Annotated in GO	13458	15.31
Annotated in KOG	7866	8.95
Annotated in all Databases	3151	3.58
Annotated in at least one Database	22722	25.86
Total Unigenes	87860	100

NR: NCBI non-redundant protein sequences. NT: NCBI nucleotide sequences. KO: Kyoto Encyclopedia of Genes and Genomes Ortholog. SwissProt: A manually annotated and reviewed protein sequence database. PFAM: Protein family. GO: Gene Ontology. KOG: euKaryotic Ortholog Groups.

Gene Ontology was used to classify the unigenes into three categories, including biological process, cellular component and molecular function. Only 13,458 unigenes (27.05%) of the transcriptome were annotated in the Gene Ontology database. In addition, one unigene potentially matched many functional groups, and the unigenes were further assigned to 1704 functional groups, including 1176 functional groups of biological process, 370 functional groups of cellular component and 158 functional groups of molecular function (Figure S2).

Annotations based on euKaryotic Ortholog Groups (KOG) of proteins were performed, and 7866 annotated unigenes (8.95%) were divided into 26 groups. Among these groups, the largest and smallest KOG group was ‘general function prediction only’ with 1673 genes and ‘unnamed protein’ with 1 gene, respectively (Figure S3).

A total of 4754 unigenes (7.68%) were annotated using the Kyoto Encyclopaedia of Genes and Genomes (KEGG) orthologue and assigned into 5 branches: Cellular Processes, Environmental Information Processing, Genetic Information Processing, Metabolism and Organismal Systems (Figure S4). Signal transduction pathway with 857 genes is the biggest pathway of KEGG classification, followed by the pathway of endocrine system (515 genes) and the pathway of translation (507 genes).

### Identification of putative apoptosis-related genes in Sf9 cells

To understand the apoptotic mechanisms of Sf9 cells, 97 putative apoptosis-related genes were identified through BLAST and KEGG orthologues in the transcriptome of Sf9 cells, including 4 members of the caspase family (*Caspase 1, 2, 5 and 6*), 4 members of the inhibitors of apoptosis (*IAP*) protein family (*IAP, IAP-2, Survivin, and Survivin-1*), 2 members of the RHG family (*IBM1 and Grim-1*9), and one member of Bcl-2 family (*Buffy*) (Table 2). Some genes, including *Sf-Caspase 1*, *Sf-Dronc*, *Sf-IAP*, *Sf-p53*, *Sf-Cathepsin B*, *Sf-Cathepsin L Sf-DnaJ1, Sf-EcR* and *Sf-TCTP*, have been reported in the past and accessed in NCBI under accession numbers U81510.1, JX912275.1, AF186378.1, HM773026.1, HQ110064.1, HQ110065.1, KF562156.1, AF411254.1 and KF562155.1, respectively, while other 88 transcripts identified in the present study were novel in Sf9 cells.

**Table 2:** Apoptosis-related genes of *Spodoptera frugiperda* in Sf9 cells.

Best Blastx Match								
Name	Gene ID	ORF (bp)	Name	Accession number	Species	Query cover (%)	NCBI E value	Identify (%)
*Sf-Aif*	c32217_g1	1602	Apoptosis-inducing factor	XM_021340279.1	*Helicoverpa armigera*	95	0	76
*Sf-Aif1*	c14745_g1	1932	Apoptosis-inducing factor 1	XM_013341813.1	*Amyelois transitella*	86	0	76
*Sf-Aif3*	c3517_g1	1782	Apoptosis-inducing factor 3	XM_021326783.1	*Helicoverpa armigera*	99	0	79
*Sf-Akt*	c71210_g1	1479	Serine/threonine protein kinase Akt	XM_021325337.1	*Helicoverpa armigera*	100	0	91
*Sf-Alg-2*	c30322_g1	540	Apoptosis-linked protein 2	EF210317.1	*Bombyx mori*	98	3e-133	80
*Sf-Apaf-1*	c12514_g1	4608	Apoptotic protease activating factor 1	XM_021325983.1	*Helicoverpa armigera*	100	0	75
*Sf-Ask1*	c32028_g1	3783	Protein kinase ASK1	XM_012693084.2	*Bombyx mori*	92	0	78
*Sf-aspp1*	c25023_g1	3198	Apoptosis-stimulating of p53 protein 1	XM_013336081.1	*Amyelois transitella*	99	0	89
*Sf-Atf2*	c22733_g1	1425	Cyclic AMP-dependent transcription factor 2	NM_001256981.1	*Bombyx mori*	34	2e-78	74
*Sf-Atf6*	c32255_g1	2346	Cyclic AMP-dependent transcription factor ATF-6	XM_021332166.1	*Helicoverpa armigera*	96	0	79
*Sf-Atm*	c24579_g1	8448	Serine-protein kinase	XM_021328438.1	*Helicoverpa armigera*	43	0	75
*Sf-Bi1*	c51885_g1	705	Bax inhibitor-1	NM_001098350.2	*Helicoverpa armigera*	100	0	88
*Sf-Bnip3L*	c9206_g1	573	Bcl2/adenovirus E1B 19 kDa protein-interacting protein 3-like	XM_011554879.1	*Plutella xylostella*	90	2e-116	78
*Sf-Buffy*	c27376_g1	876	Bcl-2-related ovarian killer protein homolog A-like	NM_001204480.1	*Helicoverpa armigera*	99	0	84
*Sf-Bun*	c30625_g1	3006	Bunched	XM_021344724.1	*Helicoverpa armigera*	99	0	90
*Sf-Cal*	c32599_g1	450	Calmodulin	HM445737.1	*Spodoptera littoralis*	100	0	99
*Sf-Cal4*	c15286_g1	345	Calmodulin-like protein 4	XM_021344204.1	*Helicoverpa armigera*	100	1e-91	82
*Sf-CaMKII*	c15582_g1	1533	Ca2+/calmodulin-dependent protein kinase II	XM_021333370.1	*Helicoverpa armigera*	100	0	88
*Sf-caspase-1*	c52422_g1	900	Cysteinyl aspartate specific proteinase-1	U81510.1	*Spodoptera frugiperda*	100	0	100
*Sf-caspase-2*	c21305_g1	837	Cysteinyl aspartate specific proteinase-2	KP711808.1	*Spodoptera frugiperda*	100	0	100
*Sf-caspase-5*	c18838_g1	1344	Cysteinyl aspartate specific proteinase-5	JX912275.1	*Spodoptera frugiperda*	100	0	100
*Sf-caspase-6*	c80794_g1	1659	Cysteinyl aspartate specific proteinase-6	KU668855.1	*Spodoptera frugiperda*	100	0	99
*Sf-CAPN7*	c31659_g9	1626	Calpain-7-like	XM_021332843.1	*Helicoverpa armigera*	100	0	81
*Sf-Cathepsin B*	c15179_g1	1026	Cathepsin B-like proteinase	HQ110064.1	*Spodoptera frugiperda*	99	0	99
*Sf-Cathepsin D*	c1417_g1	1155	Cathepsin D	KX827245.1	*Spodoptera exigua*	100	0	94
*Sf-Cathepsin L*	c32701_g1	1035	Cathepsin L-like proteinase	HQ110065.1	*Spodoptera frugiperda*	100	0	99
*Sf-Cathepsin O*	c30616_g3	1122	Cathepsin O	XM_021343406.1	*Helicoverpa armigera*	83	2e-178	76
*Sf-Cdc2*	c80616_g1	960	Cell division cycle 2	FJ157364.1	*Galleria mellonella*	95	0	81
*Sf-Cna*	c42967_g1	1488	Calcineurin A	KR185962.1	*Helicoverpa armigera*	100	0	96
*Sf-Cnb*	c71240_g1	513	Calcineurin B	FJ555522.1	*Heliothis virescens*	100	0	93
*Sf-Crea*	c22361_g2	1125	Cyclic AMP response element binding protein A	XM_021336273.1	*Helicoverpa armigera*	100	0	85
*Sf-Creb*	c29193_g1	798	Cyclic AMP response element-binding protein B	XM_021325533.1	*Helicoverpa armigera*	100	0	88
*Sf-Cyt c*	c80536_g1	327	Mitochondrial cytochrome c	JX313131.1	*Spodoptera litura*	100	2e-152	97
*Sf-Dapk*	c22942_g1	1302	Death-associated protein kinase related-like	XM_011556525.1	*Plutella xylostella*	88	0	78
*Sf-Dff*	c26660_g2	1074	DNA fragmentation factor subunit beta-like	XM_021337193.1	*Helicoverpa armigera*	100	0	85
*Sf-DnaJ1*	c30990_g1	1212	DnaJ homolog subfamily A member 1	KF562156.1	*Spodoptera frugiperda*	100	0	99
*Sf-EcRA*	c30842_g5	1545	Ecdysone receptor A	GU296540.1	*Spodoptera exigua*	100	0	95
*Sf-Endo G*	c3693_g1	2229	Flap endonuclease GEN	XM_021345082.1	*Helicoverpa armigera*	99	0	76
*Sf-Erk*	c71654_g1	1095	Extracellular regulated MAP kinase	NM_001043456.1	*Bombyx mori*	99	0	81
*Sf-Ero1*	c25409_g1	1506	Endoplasmic reticulum oxidoreduction 1-like	XM_021329593.1	*Helicoverpa armigera*	100	0	86
*Sf-FAF1*	c31337_g1	1929	Fas-associated factor 1	XM_021327322.1	*Helicoverpa armigera*	100	0	84
*Sf-FoxK*	c9138_g1	1644	Forkhead box K1	XM_021336910.1	*Helicoverpa armigera*	100	0	87
*Sf-FoxO*	c29425_g1	1530	Forkhead box sub-group O	KP735963.1	*Helicoverpa armigera*	100	0	87
*Sf-FEN-1*	c31489_g1	1143	Flap endonuclease-1	XM_013282436.1	*Papilio polytes*	100	0	77
*Sf-Gadd45a*	c788_g1	498	Growth arrest and DNA damage-inducible, alpha	XM_021341866.1	*Helicoverpa armigera*	100	4e-150	84
*Sf-Ghitm*	c1686_g1	1005	Growth hormone-inducible transmembrane	XM_021335632.1	*Helicoverpa armigera*	100	0	80
*Sf-Grim19*	c72166_g1	459	Protein-like Grim-19	HM369463.1	*Helicoverpa armigera*	96	3e-163	89
*Sf-Gsk3*	c8059_g1	1248	Glycogen synthase kinase-3	KJ206238.1	*Helicoverpa armigera*	74	0	95
*Sf-Hsp70*	c25533_g2	1962	70 kDa heat shock protein	KC787696.1	*Spodoptera littoralis*	100	0	97
*Sf-Iap*	c51844_g1	1134	Inhibitor of apoptosis protein	AF186378.1	*Spodoptera frugiperda*	100	0	99
*Sf-Iap2*	c32032_g11	1863	Inhibitor of apoptosis protein 2	NM_001202529.1	*Bombyx mori*	66	2e-98	68
*Sf-Ibm1*	-	285	IAP-binding motif 1	NM_001166341.1	*Lymantria dispar*	100	8e-118	94
*Sf- Ikbka*	c18461_g2	447	Inhibitor of nuclear factor kappa-B kinase subunit alpha	XM_011568209.1	*Plutella xylostella*	100	6e-71	74
*Sf- Ikbkb*	c18461_g1	1845	Inhibitor of nuclear factor kappa-B kinase subunit beta	XM_004928112.1	*Bombyx mori*	91	6e-148	68
*Sf-Jnk*	c31936_g1	1143	c-Jun NH2-terminal kinase	JF727877.1	*Helicoverpa armigera*	92	0	91
*Sf-Jip3*	c28014_g1	2619	c-Jun NH2-terminal kinase-interacting protein 3	XM_021332213.1	*Helicoverpa armigera*	93	0	86
*Sf-Litaf*	c14041_g1	309	LPS-induced TNF-alpha factor	XM_021330760.1	*Helicoverpa armigera*	40	2e-05	74
*Sf-Mam*	c80524_g1	2220	Mastermind, activator of Notch signalling	XM_004927862.1	*Bombyx mori*	100	0	77
*Sf-Map2k7*	c25597_g1	2034	Mitogen-activated protein kinase kinase 7	XM_021339636.1	*Helicoverpa armigera*	93	0	85
*Sf-Map3k4*	c31077_g4	4425	Mitogen-activated protein kinase kinase kinase 4	XM_021337467.1	*Helicoverpa armigera*	89	0	80
*Sf-Map3k7*	c29754_g1	1971	Mitogen-activated protein kinase kinase kinase 7	XM_021342758.1	*Helicoverpa armigera*	71	0	82
*Sf-Map4k5*	c28599_g3	3324	Mitogen-activated protein kinase kinase kinase kinase 5	XM_021341817.1	*Helicoverpa armigera*	94	0	81
*Sf-Map2k4*	c80780_g1	1236	Mitogen-activated protein kinase kinase 4	XM_021334598.1	*Helicoverpa armigera*	100	0	90
*Sf-Map2k6*	c42264_g1	1002	Mitogen-activated protein kinase kinase 6	XM_021337042.1	*Helicoverpa armigera*	100	0	89
*Sf-Nup53*	c24775_g1	906	Nucleoporin *NUP53*	XM_021332320.1	*Helicoverpa armigera*	100	0	82
*Sf-p53*	c31241_g2	1125	p53	HM773026.1	*Spodoptera frugiperda*	100	0	99
*Sf-Parp*	c61913_g1	2997	poly [ADP-ribose] polymerase-like	XM_011562810.1	*Plutella xylostella*	98	0	67
*Sf-Pcdp2*	c27093_g1	1224	Programmed cell death protein 2	XM_021340263.1	*Helicoverpa armigera*	97	0	81
*Sf-Pcdp4*	c32143_g3	1350	Programmed cell death protein 4	XM_004925107.3	*Bombyx mori*	99	0	77
*Sf-Pcdp5*	c9286_g1	393	Programmed cell death protein 5	XM_021325942.1	*Helicoverpa armigera*	100	6e-115	84
*Sf-Pcdp10*	c23215_g2	630	Programmed cell death protein 10	XM_001657732.2	*Aedes aegypti*	98	3e-70	70
*Sf-Pcdp11*	c30865_g7	3747	Programmed cell death 11-like protein	JF681972.1	*Spodoptera litura*	35	0	90
*Sf-Pdi*	c30611_g1	1485	Protein disulfide isomerase	JX183988.1	*Spodoptera litura*	100	0	95
*Sf-Pdrg*	c31235_g1	399	p53 and DNA damage-regulated protein	XM_013289208.1	*Papilio polytes*	89	5e-27	69
*Sf-Pi3k*	c254_g1	1200	Phosphatidylinositol 3-kinase 60	NM_001127721.1	*Bombyx mori*	99	3e-166	71
*Sf-Pik3c3*	c22894_g1	2763	phosphatidylinositol 3-kinase catalytic subunit type 3	XM_021338589.1	*Helicoverpa armigera*	99	0	79
*Sf-Pkac1*	c80575_g1	1062	cAMP-dependent protein kinase C1	KT207930.1	*Helicoverpa armigera*	100	0	93
*Sf-Pkar1*	c62273_g1	1119	cAMP-dependent protein kinase R1	NM_001099825.1	*Bombyx mori*	100	0	77
*Sf-Pkar2*	c80500_g1	1155	cAMP-dependent protein kinase R2	NM_001111353.1	*Bombyx mori*	100	0	85
*Sf-Pkdc2*	c23609_g1	1089	Protein kinase DC2	XM_021339075.1	*Helicoverpa armigera*	100	0	87
*Sf-p38 Mapk*	c25060_g1	1083	p38 map kinase	KC895497.1	*Helicoverpa armigera*	100	0	87
*Sf-Rab39b*	c25750_g1	669	Ras-related protein Rab-39B	XM_021328859.1	*Helicoverpa armigera*	100	0	89
*Sf-Ras*	c25394_g1	555	Ras protein	NM_001043507.1	*Bombyx mori*	89	5e-137	82
*Sf-Ras2*	c23015_g1	603	Ras protein 2	XM_021335345.1	*Helicoverpa armigera*	100	0	88
*Sf-Rock1*	c12286_g1	4080	Rho-associated protein kinase1	XM_013278227.1	*Papilio polytes*	100	0	80
*Sf-S6k*	c26743_g1	1362	Ribosomal protein S6 kinase	KU987819.1	*Helicoverpa armigera*	99	0	86
*Sf-Rps6ka5*	c28599_g2	3819	Ribosomal protein S6 kinase alpha 5	XM_021341820.1	*Helicoverpa armigera*	84	0	80
*Sf-Rptor*	c25945_g1	747	Regulatory-associated protein of mTOR	XM_021341810.1	*Helicoverpa armigera*	90	0	89
*Sf-Stat*	c31032_g9	2307	signal transducer and activator of transcription	AF329946.1	*Spodoptera frugiperda*	100	0	99
*Sf-Survivin*	c28129_g1	420	Apoptosis inhibitor survivin	DQ875271.2	*Helicoverpa armigera*	99	2e-90	79
*Sf-Survivin-1*	c17897_g1	4029	Apoptosis inhibitor survivin-1	XM_012688679.2	*Bombyx mori*	84	0	78
*Sf-Tgfbr1*	c28315_g2	1185	TGF-beta receptor type-1	KY328721.1	*Helicoverpa armigera*	97	0	84
*Sf-TCTP*	c9564_g1	519	Translationally controlled tumor protein	KF562155.1	*Spodoptera frugiperda*	100	0	100
*Sf-Tnfsf5*	c28372_g1	1017	TNFSF5	XM_012693655.2	*Bombyx mori*	100	6e-126	70
*Sf-Traf3*	c24205_g1	777	TNF receptor-associated factor 3	NM_001256987.1	*Bombyx mori*	99	2e-105	71
*Sf-Traf4*	c23191_g1	1404	TNF receptor-associated factor 4	XM_021325240.1	*Helicoverpa armigera*	100	0	86
*Sf-Traf6*	c29008_g2	1254	TNF receptor-associated factor 6	XM_021327070.1	*Helicoverpa armigera*	100	0	84

### Caspase family members in Sf9 cells

**Figure 1:**
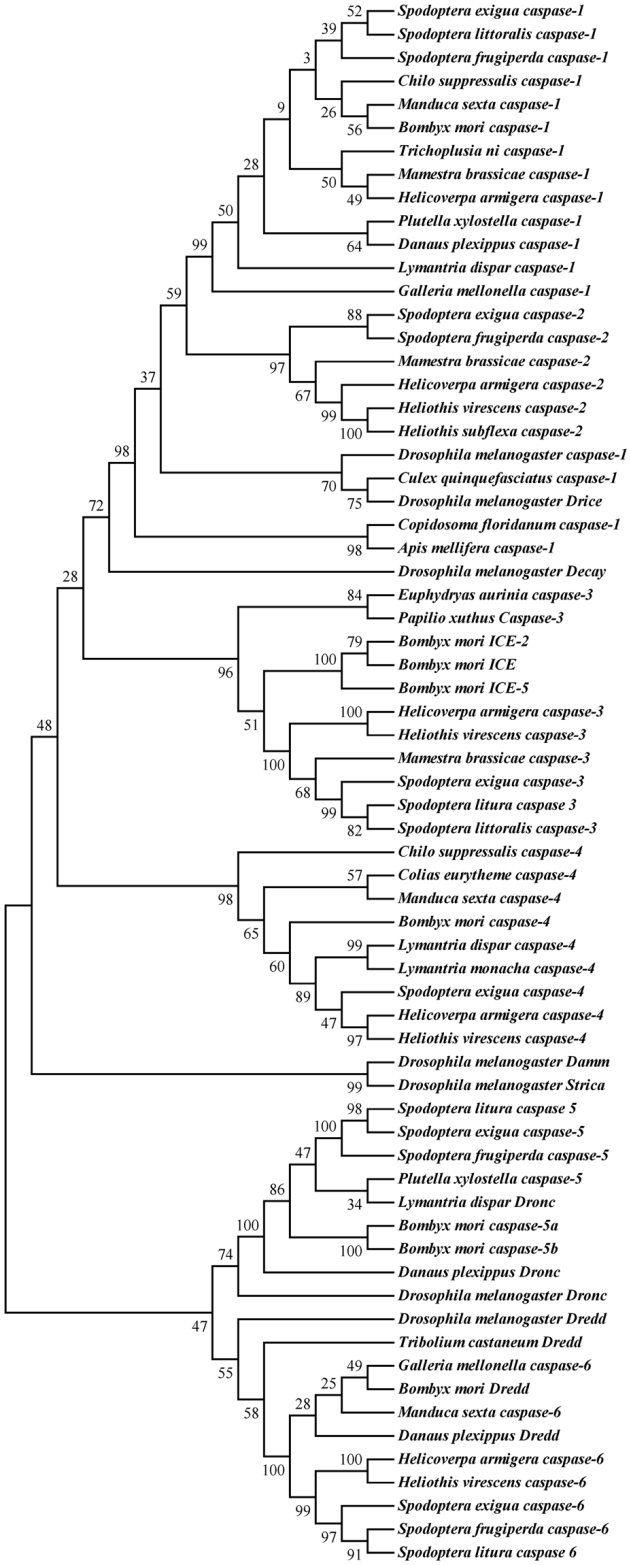
Phylogenetic relationships between caspase sequences observed in Sf9 cells transcriptome and insects from the NCBI database. Phylogenetic analyses were performed using MEGA version 5.0 based on the amino acid sequences. Values indicated at the nodes are bootstrap values based on 1000 replicates.

Caspases are a family of intracellular cysteine proteases that play vital roles in apoptosis. These proteins can be classified as initiator and effector caspases containing three different regions: one N-terminal prodomain, one large catalytic subunit (p20) and one small catalytic subunit (p10)^31^. Four caspases were identified from the transcriptome of Sf9 cells, including two initiators (c18838_g1 and c80794_g1) and two effectors (c52422_g1 and c21305_g1). According to the classification criteria of lepidoptera insect caspases and the results of BLAST comparative analysis, four caspases were identified: *Sf-Caspase 5*, *Sf-Caspase 6*, *Sf-Caspase 1* and *Sf-Caspase 2*. Protein sequence analysis demonstrated that all of these enzymes had a highly conserved five-peptide sequence of QACXG (X for R, Q or G), similar to most caspases^32^. Additionally, a phylogenetic tree of the caspases in Sf9 cells and other insects was constructed (Fig. 1), showing a close relationship between *S*. *frugiperda* caspases and lepidopteran caspases.

### Inhibitors of apoptosis (IAP) protein family members

**Figure 2:**
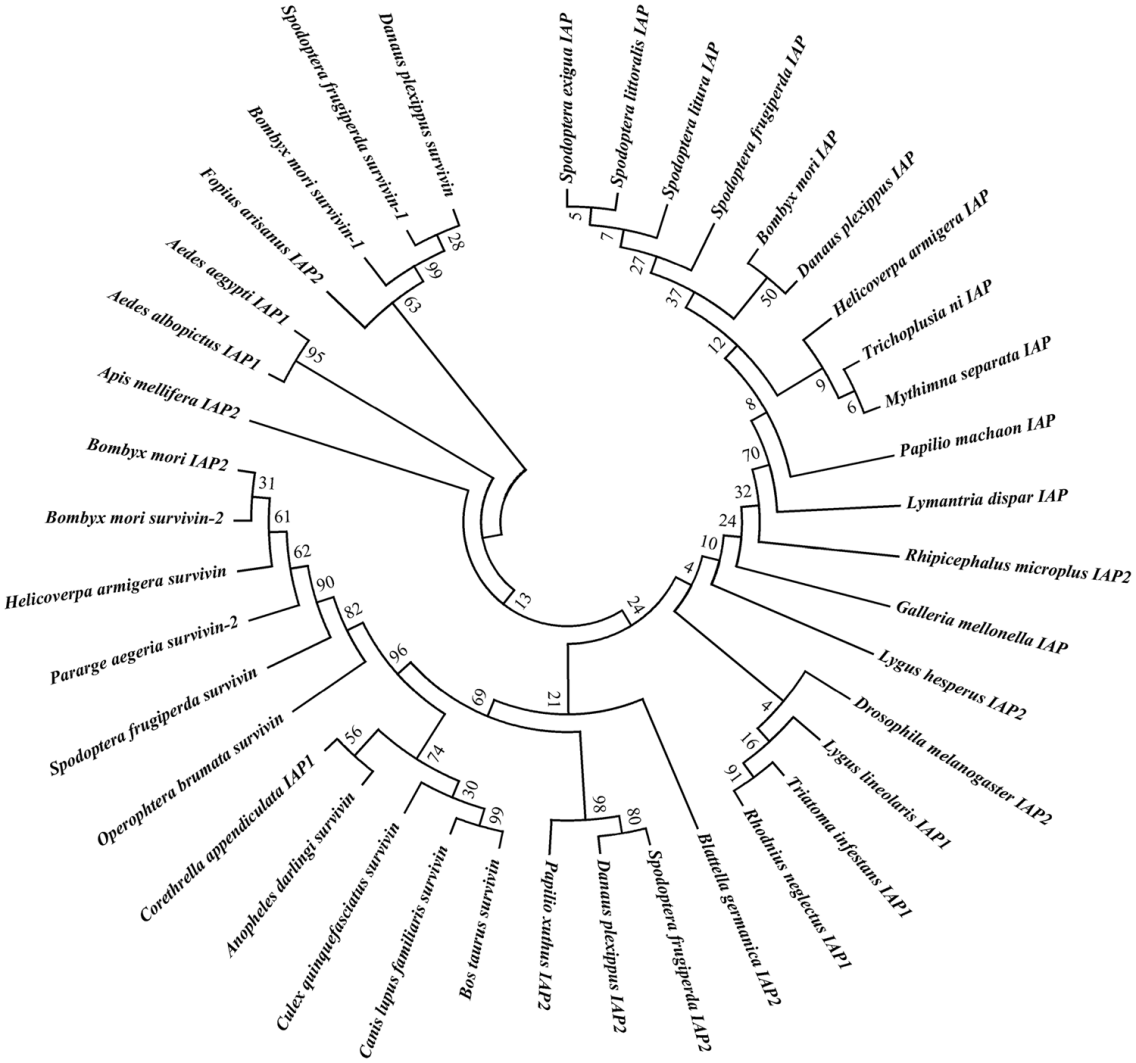
Phylogenetic relationships between IAP sequences observed in Sf9 cells transcriptome and insects from NCBI database. The tree was constructed with MEGA 5.0 using the neighbour-joining method.

The IAP family was initially identified in insect baculoviruses, and these proteins play important roles in regulating apoptosis through binding to and inhibiting the activity of caspases^16,33^. Four members of the IAP family, c51844_g1, c32032_g11, c28129_g1 and c17897_g1, were identified in transcriptome, and sequence alignment analysis indicated that these proteins were *Sf-IAP*, *Sf-IAP-2*, *Sf-Survivin* and *Sf-Survivin-1*. The four IAP family members were divided into two types based on their different structures. *Sf-IAP* and *Sf-IAP-2* proteins belong to type I IAPs, where *Sf-IAP* contains two BIR (baculoviral inhibitor of apoptosis repeat) domains and *Sf-IAP-2* contains three BIR domains. Additionally, both genes possess a RING finger domain in the carboxyl-terminus identified as an ubiquitin-conjugating enzyme^16^. *Sf-Survivin* and *Sf-Survivin-1* are characterized as another type of IAP, which only has one BIR repeat. The phylogenetic tree of the four IAP family members was constructed using MEGA 5.0 (Fig. 2).

### RHG family members

As IAP antagonists, RHG family members, such as reaper, hid and grim are primary regulators in controlling programmed cell death in insects^34^. In the transcriptome of Sf9 cells, we discovered two RHG genes, identified as *Sf-IBM1* (IAP-binding motif 1) and *Sf-Grim-19* by blasting to the NCBI. *Sf-IBM1* has a 258-bp putative open reading frame (ORF), encoding a polypeptide of 94 amino acid residues with a predicted molecular mass of 10.81 kDa. The molecular mass of *Sf-Grim-19* protein with 152 amino acids was 45.45 kDa with an isoelectric point of 9.42. BLAST alignments showed that *Sf-IBM1* had high homology with IAP-binding motif 1 in *B*. *mori* and *P*. *xylostella*, and the nucleotide similarities were 86% and 79%, respectively. Few grim genes have been identified in lepidoptera insects. However, we observed that *Sf-Grim-19* was similar to grim-19 in *Helicoverpa armigera* with 86% nucleotide identity. Multiple sequence alignments of both genes have been accomplished as shown in Figure S5.

### Bcl-2 family member: Sf-Buffy

As anti-apoptotic proteins, the Bcl-2 family regulates apoptosis through both direct and indirect interactions with p53^35^. Few Bcl-2 family members have been identified or previously reported in lepidoptera. In the present study, we identified a member of the Bcl-2 family: *Sf-Buffy*. The length of the *Sf-Buffy* ORF was 876 bp, encoding a protein with 291 amino acids. Protein structure analysis revealed that *Sf-Buffy* had four conserved BH domains (BH1, BH2, BH3 and BH4) and one BH3-homology binding site region. BLASTP alignments indicated that *Sf-Buffy* shared highly similar amino acid sequences with *Danaus plexippus* (84%) and *Bombyx mori* (81%).

### Most of the putative apoptosis-related genes are actively transcribed in Sf9 cells

**Figure 3:**
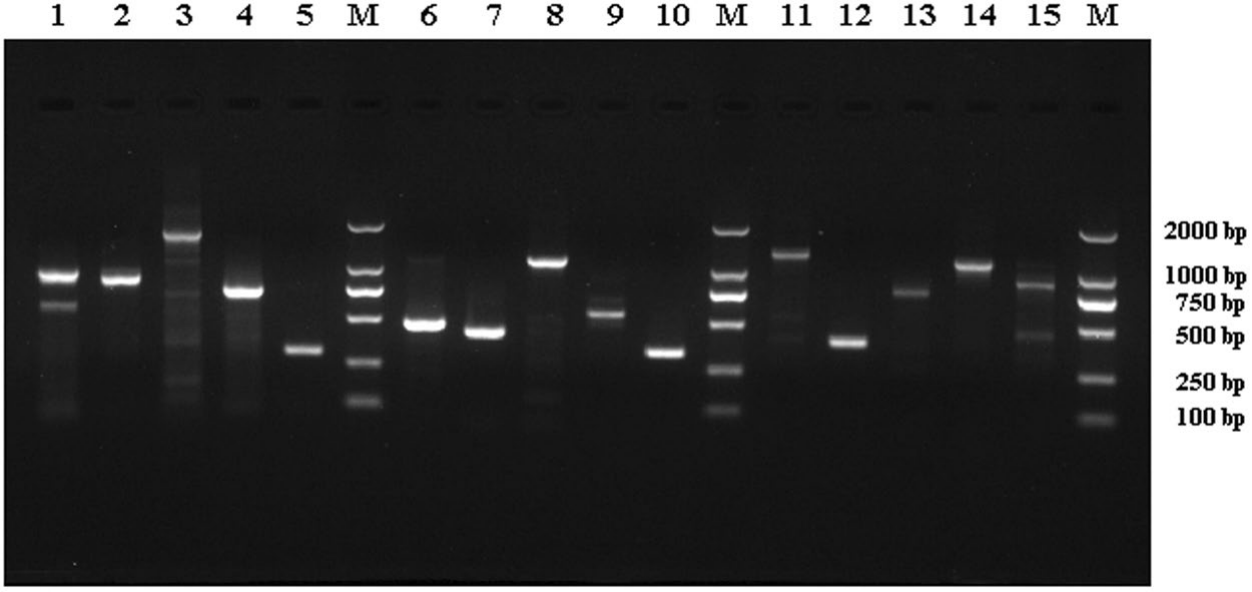
Agarose gel of some apoptosis-related genes in *S. frugiperda* PCR amplified with the specific primers shown in Supplement Table 1. Lanes: M, Marker 2000, Lane 1–15, *Sf-Caspase-1*, *Sf-Caspase-2*, *Sf-AIF*, *Sf-BI*, *Sf-IBM1*, *Sf-Grim-19*, *Sf-Survivin*, *Sf-IAP*, *Sf-Ras*, *Sf-Cyt C*, *Sf-Traf 6*, *Sf-Pcdp 5*, *Sf-Rptor*, *Sf-Pkar 1*, and *Sf-Buffy*.

As shown in Fig. 3, 15 representative PCR products were examined using agarose gel electrophoresis, and the lengths of the PCR fragments were consistent with the sizes of gene-encoding region predicted in the transcriptome analysis. The genes considered the critical factors of apoptosis, such as the main effectors *Sf-caspase-1* and *Sf-caspase-2* and the key genes in the mitochondrial apoptotic pathway including *Sf-Buffy*, *Sf-Cytochrome c*, *Sf-IAP* and *Sf-Bax inhibitor*, were amplified. In addition, the pro-apoptosis genes *Sf-Grim-19*, *Sf-IBM1* and other genes involved in various signal pathways were successfully amplified. The successful amplification and identification of the coding region of key apoptosis-related genes indicated that the typical apoptotic pathways were present in Sf9 cells, and the apoptosis-related genes were actively transcribed in Sf9 cells. Moreover, these results also showed the conservation of apoptosis pathways between *S*. *frugiperda* and the lepidopteran insects.

### mRNA expression profile of the main putative apoptosis-related genes in apoptosis induced by azadirachtin

**Figure 4:**
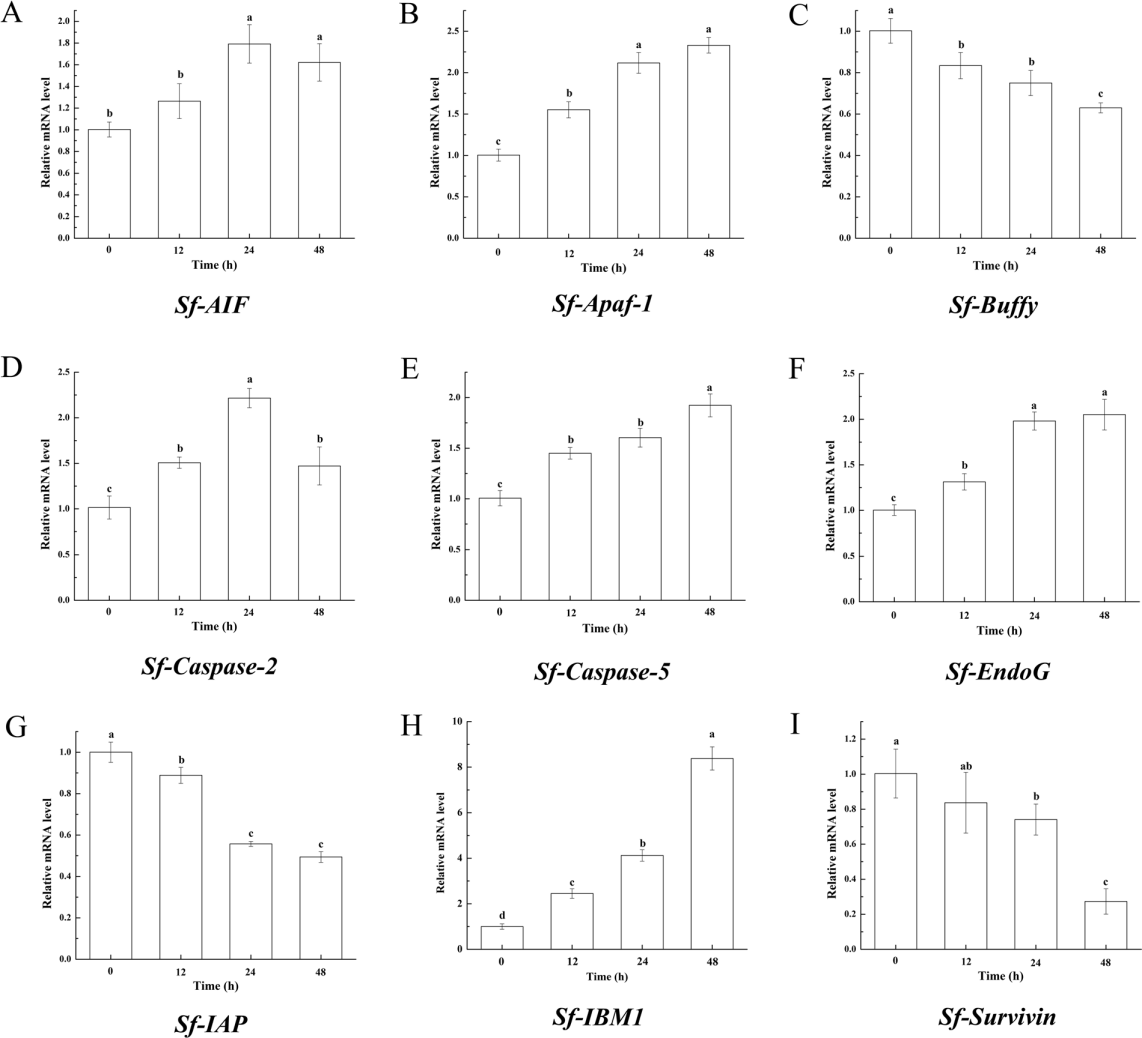
The qRT-PCR analysis of 9 apoptosis-related genes between controls and cells treated with azadirachtin at 12, 24 and 48 h. The GAPDH gene was used as the housekeeping gene, and the data are expressed as arithmetic mean ± SEM (n = 3). Different letters above bars indicate significant differences between different treatments (P < 0.05) using ANOVA, followed by DMRT.

To determine the action of azadirachtin at the transcriptional level, quantitative RT-PCR was used to confirm the mRNA expression levels of 10 genes that play important roles in caspase-dependent or caspase-independent pathways. As shown in Fig. 4, the mRNA expression levels of 7 genes involved in the caspase-dependent apoptotic pathway were significantly different between controls and azadirachtin-treated cells at different intervals. The levels of *Sf-Apaf-1*, *Sf-Caspase-2*, *Sf-Caspase-5* and *Sf-IBM1* increased, while the levels of *Sf-Buffy*, *Sf-IAP* and *Sf-Survivin* decreased. After exposure to 0.75 μg/mL azadirachtin for 48 h, the mRNA expression of *Sf-Apaf-1*, *Sf-Caspase-5* and *Sf-IBM1* increased 133%, 92.3% and 651.3%, respectively (Fig. 4B,H and E). Similarly, the mRNA expression of *Sf-Caspase-2* increased 121.7% after exposure to 0.75 μg/mL azadirachtin for 24 h. In addition, 37.1%, 50.6% and 72.7% decreases in the mRNA expression of *Sf-Buffy*, *Sf-IAP* and *Sf-Survivin* were observed after treatment with azadirachtin for 48 h (Fig. 4C,G and H). We also observed that the mRNA expression of *Sf-AIF1* and *Sf-EndoG* which associated with the caspase-independent apoptotic pathway, as the mRNA expression of *Sf-AIF1* increased 79.2% after azadirachtin treatment for 24 h and the mRNA expression of *Sf-EndoG* increased 105.1% of after azadirachtin treatment for 48 h (Fig. 4A and Fig. 4F). Additionally, we observed that *Sf-Cytochrome c* showed almost no significant change after azadirachtin treatment (Figure S6). Analysis of the gene expression after azadirachtin treatment showed that azadirachtin induced apoptosis through caspase-dependent or caspase-independent apoptotic pathways at the transcriptional level.

### Effect of azadirachtin on the putative apoptosis-related protein levels in Sf9 cells

**Figure 5:**
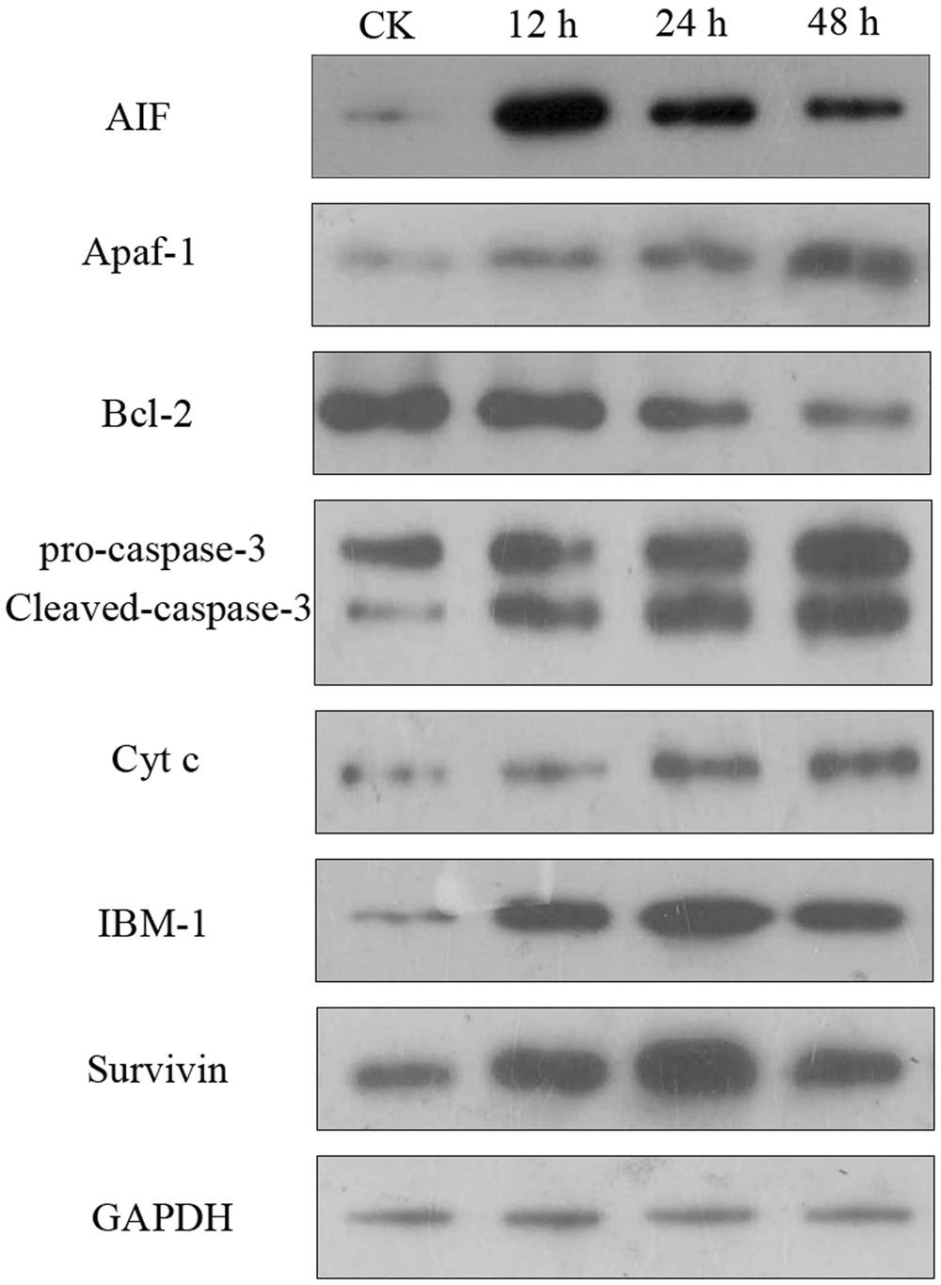
Western blot analysis of seven apoptosis-related proteins between controls and cells treated with azadirachtin at 12, 24 and 48 h. The GAPDH was used to normalize the difference.

In order to further confirming that azadirachtin induced apoptosis through caspase-dependent or caspase-independent apoptotic pathway, the effects of azadirachtin on proteins considered as the key factors of apoptosis signal pathway were detected by western blot. As shown in Fig. 5, the expression of cytochrome c, bcl-2, Apaf-1 and IBM1 proteins involved in caspase-dependent apoptosis pathway were changed after azadirachtin treatments in a time-dependent manner. There was an obviously decrease of bcl-2 expression levels while the protein levels of cytochrome c, Apaf-1 and IBM1 increased after azadirachtin treatment. Simultaneously, the protein levels of cleaved caspase-3 was increased significantly in azadirachtin treatment samples. As expected, an obvious increase of AIF protein level was observed after azadirachtin treatment. In addition, Survivin plays roles as an apoptosis inhibitor and the protein expression level of Survivin were increased after azadirachtin treatments. These results confirmed that these proteins were involved in the process of apoptosis induced by azadirachtin in S9 cells.

## Discussion

With continuous development, high-throughput sequencing technologies have become the conventional means for biological studies. Recently, the genomic resource of *S*. *frugiperda* has been revealed. A draft genome sequence and transcriptome were assembled by *de novo* sequencing of genomic DNA and mRNA from Sf21 cells (a cell line derived from the ovaries of *S*. *frugiperda*)^36,37^. Additionally, a reference transcriptome for *S*. *frugiperda* was constructed from *S*. *frugiperda* samples, which contained different developmental time-points and tissues, using NGS^1^. Furthermore, the transcriptome of Sf9 was obtained, and the insecticidal mechanisms of AcMNPV-BmK IT and AcMNPV treatment have been explained^38^. In the present study, we assembled the transcriptome of the *S*. *frugiperda* cell line Sf9 and observed that the data of this transcriptome were different from that of Legeai 2014 and Wei 2017, and more sequence numbers were observed in the transcriptome delineated in the present study (Supplement Table 2), which could be explained by the diversity of insect transcriptomes in each cell type, tissue and organ system^39^ and the different sequencing techniques employed.

Apoptosis is an important and complex physiological process involving many factors. The identification and analysis of putative apoptosis-related genes play important roles in elucidating apoptotic mechanisms. For example, the cloned *Sf-IAP* had a similar amino acid sequence and evolutionary conserved function compared with the baculoviral IAPs^40^. In addition, the transgenic expression of *Sf-IAP* in plants inhibits the programmed cell death induced under several selection pressures, including heat, salt and fumonisin B1 (FB1). The E3 ubiquitin ligase activity of a RING finger at the carboxyl-terminus in *Sf-IAP* is essential for stress protection in plants^41,42^. p53 is a tumour suppressor that has been extensively studied. *Sf-p53* contains 374 amino acids with a predicted molecular mass of 42.5 kDa. Additionally, overexpression of *Sf-p53* in Sf9 cells induced apoptosis and caspase activities^43^. Despite the pivotal roles of caspases in apoptosis and *S*. *frugiperda* cell lines as a model for apoptosis research, only two caspase genes (*Sf-Caspase-1* and *Sf-Dronc*) have previously been reported. As the principal effector caspase, *Sf-Caspase-1* was identified with 299 amino acids and a predicted molecular weight of 35 kDa. Studies have shown that *Sf-Caspase-1* induces apoptosis and cleaves nuclear immunophilin FKBP45 in Sf9 cells^44^. *Sf-Dronc* was identified as an initiator caspase that cleaves and activates the effector caspase *Sf-Caspase-1*. Furthermore, apoptosis occurred in Sf9 cells through the overexpression of Sf-Dronc^45^. The caspases in lepidoptera are classified into 6 distinct classes (caspase-1 to −6) based on phylogenetic analyses^46^. The four orthologous caspases identified in the present study were divided into putative initiator caspases [*Sf-Caspase-5* (*Sf-Dronc*) and *Sf-Caspase-6* (*Sf-Dredd*)] and putative effector caspases (*Sf-Caspase-1* and *Sf-Caspase-2*). *Sf-Dredd* is the homologue of dipteran Dredd and overexpression could induced apoptosis in Sf9 cells, but the effectiveness was less than *Sf-Dronc* ^47^. Additionally, it is likely involved in immune response to infections with Gram-negative bacteria^48^. *Sf-Caspase-1* and *Sf-Caspase-2* have high amino acid identity, but the cleavage sites of *Sf-Caspase-1* (TETD) and *Sf-Caspase-2* (AETD) are different. Gene and function duplication could occur in these two genes that may have different signals of *Sf-caspase-2* activation^46^. Concurrently, with two identified members of the RHG family, *Sf-IBM1* and *Sf-Grim-19*, the high homology of *Bm-IBM1* and *Ha-Grim-19* indicated that these genes have the same functions in different species. Since no orthologues of caspase-3, caspase-4, hid and bruce have been identified in Sf9 cells, we hypothesized that the low expression of these four genes (caspase-3, caspase-4, hid and bruce) leads to failures of assembly and detection or these genes could be lost in evolution.

In the present study, 97 putative apoptosis-related genes in Sf9 cells were identified, and almost all of the genes were essential parts of various apoptosis pathways. As the center of intrinsic apoptosis pathway, mitochondria determines the cells fate^49,50^. The annotation of homologues of cytochrome c, AIF (apoptosis-inducing factor), apaf-1, caspases and EndoG in Sf9 cells and the evidence of azadirachtin-induced cytochrome c release in Sf9 cells proved by Huang *et al*.^28^ illustrated that the mitochondrial apoptotic pathway existed in Sf9 cells and could be one of the primary functional pathways. At the same time, we further elucidated the changes of key nodes in mitochondrial apoptotic pathways induced by azadirachtin in molecular biology methods and the results revealed the apoptosis mechanism of azadirachtin by regulating the caspase-dependent or caspase-independent apoptotic pathway to induce apoptosis in Sf9 cells. Interesting, there is no changes in mRNA expression but significant protein level changes of cytochrome c after azadirachtin treatment, suggested that the azadirachtin-induced changes in cytochrome c could be mediated through translational regulation. At the same time, we found a strange phenomenon with *Sf-Survivn* that a decrease of mRNA level and an increase of protein level were observed by *Sf-Survivn* after azadirachtin treatments, more and compelling evidence were needed to confirm its exact role.

Moreover, the identification of the cathepsin family and evidence from previous studied suggested that the lysosomal pathway plays a critical role in the apoptosis of Sf9 cells^29^. In addition, the existence of Traf family members (Traf 3, Traf 4 and Traf 6) and Fas-associated factor 1(*Sf-FAF1*) suggesting that the death receptor pathway could be functional in Sf9 cells. We also propose that the PI3K/Akt-PAK1 signaling pathway is likely present in Sf9 cells because of the identification of orthologues of PAK (cAMP-dependent protein kinase C1, R1, R2), *Sf-Creba*, *Sf-Pi3k*, *Sf-Pik3c3*, *Sf-Akt*, Ras family members (Ras and Ras 2), etc. Furthermore, *Sf-p53*, *Sf-CAPN7*, *Sf-Ero1*, *Sf-Jnk*, *Sf-Dff* involved in p53 signaling pathway, endoplasmic reticulum pathway, JNK signaling pathway and DNA damage pathway were annotated. Therefore, we suspected that intrinsic and extrinsic apoptotic pathways existed in the *S*. *frugiperda*, and the apoptotic pathways were conserved between mammals and insects.

In summary, the present study provided the transcriptome of Sf9 cells using “Next-generation” sequencing technology. Over 48 million clean reads were obtained and assembled into 87,860 unigenes. In addition, 22,722 unigenes were annotated into at least one database, with 97 putative apoptosis-related genes identified through BLAST analysis and 15 typical genes identified through PCR, suggesting that the apoptosis signaling pathways existed in Sf9 cells, which were highly conserved during evolution in insects. Conversely, the mRNA and protein expression level changes in some crucial genes examined after azadirachtin treatment at various time intervals indicated that caspase-dependent or caspase-independent apoptotic pathways could participate in apoptosis induced through azadirachtin treatment in Sf9 cells. The overview of putative apoptosis-related genes in Sf9 cells contributed to the study of the apoptosis signaling network and provided new evidence on the mechanism of apoptosis induction through azadirachtin. Furthermore, a large amount of sequence data not only enriched the biological information and diversity of the transcriptome in *S*. *frugiperda*, but also provided a general sequence resource for further molecular research of *S*. *frugiperda*.

## Materials and Methods

### Chemicals

Azadirachtin (95% purity) was purchased from Sigma. Dimethyl sulfoxide (DMSO) was purchased from Sigma and used as a solvent to dissolve azadirachtin. Grace’s insect cell culture medium was obtained from Thermo Scientific (USA), and fetal bovine serum (FBS) was purchased from Gibco (Australia). Rabbit polyclonal anti- Apaf-1, Bcl-2, survivin were purchased from Boster Biological Technology (China), Rabbit polyclonal anti-Cleaved Caspase-3 was purchased from Cell Signaling Technology (Beverly, MA, USA). Rabbit polyclonal anti-AIF and mouse polyclonal anti-Cyt c were purchased from Beyotime Biotechnology (China). The Rabbit polyclonal anti-*Sf-IBM1* was prepared by our laboratory.

### Cell Culture

Sf9 cells were obtained from School of Life Sciences, Central China Normal University (Wuhan, China) and maintained at 27 °C in 25-cm^2^ culture flasks (Nest) containing 3 mL of Grace’s insect cell culture medium enriched with 10% FBS (Gibco). The cells were subcultured every 3 days.

### Total RNA isolation and cDNA library preparation

Approximately 5 × 10^6^ Sf9 cells were collected, and the total RNA was isolated following the manufacturer’s instructions using TRIzol reagent (Invitrogen, USA). RNA degradation and contamination were assessed using 1% agarose gels. RNA purity was detected using the NanoPhotometer^®^ spectrophotometer (IMPLEN, CA, USA). An RNA Nano 6000 Assay Kit was used to assess the RNA integrity using the Agilent Bioanalyzer 2100 system (Agilent Technologies, USA). The total RNA of three biological replicates was mixed together, and 3 μg of mixed RNA was used for the RNA sample preparation. Sequencing libraries were generated using NEBNext^®^ Ultra™ RNA Library Prep Kit for Illumina^®^ (NEB, USA) following the manufacturer’s instructions.

### Sequencing and de novo assembly

These works were accomplished by the company of Novogene (China). The clustering of the index-coded samples was performed on a cBot Cluster Generation System using TruSeq PE Cluster Kit v3-cBot-HS (Illumina) according to the manufacturer’s instructions. After cluster generation, the library preparations were sequenced on an Illumina HiSeq platform. The clean data (clean reads) were obtained from raw data (raw reads) by removing reads containing adapter, reads containing poly-N and low-quality reads. In addition, error rate, Q20, Q30 and GC-content of the clean data were calculated. Subsequently, the Trinity (Version: v2012-10-05) was adopted to fulfil the transcriptome assembly following manufacturer’s instructions^51^.

### Functional analysis of Unigenes

Seven databases, including NR (NCBI non-redundant protein sequences), NT (NCBI nucleotide sequences), KOG/COG (Clusters of Orthologous Groups of proteins), Swiss-Prot (A manually annotated and reviewed protein sequence database), PFAM (Protein family), GO (Gene Ontology), and KEGG (Kyoto Encyclopaedia of Genes and Genomes), were used to annotate the whole unigenes. NCBI blast 2.2.28 + was applied for annotation in the Nr, Nt, and Swiss Prot databases with an E-value of 1e^−5^ and in KOG with an E-value of 1e^−3^. GO functional annotation was based on the results of NR and PFAM protein annotation, and the Blast2GO v2.5 was adopted using an online service at http://www.geneontology.org ^52^. The KEGG annotation was accomplished using KAAS (KEGG Automatic Annotation Server) through http://www.genome.jp/kegg/.

### Identification of putative apoptosis-related genes in the transcriptome of Sf9 cells

Putative apoptosis-related genes in Sf9 cells were certified, including caspase family, IAP family, and RHG family genes. The nucleotide sequences of the genes were obtained from the transcriptome results using the Novo finder software. Phylogenetic analyses were performed using MEGA version 5.0 based on the amino acid sequences in Sf9 cells and all Lepidoptera in the NCBI database, respectively. Multiple sequence alignments were executed using the http://multalin.toulouse.inra.fr/multalin/multalin.html website.

### Cloning of some key putative apoptosis-related genes by RT-PCR

The extracted total RNA of Sf9 cells was reverse transcribed into cDNA using the PrimeScript^TM^ II 1st Strand cDNA Synthesis Kit (TaKaRa, Japan) according to the manufacturer’s instructions. Primer pairs of the putative apoptosis-related genes were designed using Primer Premier 5.00 software according to the sequences from the transcriptome and are listed in Supplement Table 1. The cDNA was used as the template to amplify the putative apoptosis-related genes by PCR with a volume of 25 μL, and the PCR was performed for 30 cycles. The PCR products were assessed using 1% agarose gels stained with EB (ethidium bromide) and sequenced using first generation sequencing technology.

### Treatments and Quantitative RT-PCR

Approximately 2 × 10^5^ Sf9 cells were seeded onto 6-well plates and cultured overnight. The next day, 0.75 *μ*g/mL azadirachtin was exposed to Sf9 cells for 12, 24 and 48 h respectively, and subsequently, total RNA was extracted using TRIzol regent (Invitrogen, USA) following the manufacturer’s instructions. RNA concentration and purity was examined using the NanoDrop 2000 spectrophotometer (USA).

To verify the effects of azadirachtin on expression pattern of apoptosis-related genes in Sf9 cells, the cDNA synthesis for Quantitative RT-PCR were performed with 1 *μ*g total RNA using the PrimeScript^TM^ RT reagent Kit (TaKaRa, Japan) following the manufacturer’s instructions in which the gDNA Eraser in the kit was used to purify the RNA. Quantitative RT-PCR was performed in triplicate using the CFX Coxnnect^TM^ Real-Time System (Bio-Rad, USA) with the SsoAdvanced^TM^ SYBR^®^ Green Supermix (Bio-Rad, USA). The thermal cycle conditions were as follows: 1 cycle (95 °C for 3 min), followed by 40 cycles (95 °C for 10 s; 61 °C for 10 s; 72 °C for 30 s), followed by 1 cycle for the dissociation stage (95 °C for 10 s; 65 °C for 5 s; 95 °C for 15 s). The expression of apoptosis-related genes was calculated using the 2^−ΔΔCt^ method^53^. GAPDH (glyceraldehyde-3-phosphate dehydrogenase) was used as the reference gene, and the primer sequences of apoptosis-related genes are listed in Supplement Table 1.

### Western blotting

Azadirachtin-treated Sf9 cells of each treatment were collected and washed with PBS twice. The protein samples were resuspended using CytoBuster^TM^ Protein Extraction Reagent (Novagen, USA) and incubated at 25 °C by shaking for 10 min and centrifugation at 14000 x *g* for 5 min at 4 °C. The supernatants were used for Western blotting, and the protein concentration was detected using the BCA protein assay kit (Tiangen, China). The same amount of protein samples was separated on a 12% SDS–PAGE gel and transferred to a PVDF membrane. After blocking in TBS with 5% fat-free milk, the membrane was incubated with specific primary antibodies, followed by incubation with secondary antibody. The enhanced chemiluminescence (ECL) method was used to visualize the protein bands, and GAPDH was used to normalize the difference.

### Data analysis

All collected data are expressed as the mean ± SD (n = 3), and Duncan’s new multiple range test (DMRT) was used to perform the statistical analysis with the statistical significance of P < 0.05.

## Electronic supplementary material

Supplementary File
