# Supplementary material for: Transcriptome analysis of Spodoptera frugiperda Sf9 cells reveals putative apoptosis-related genes and a preliminary apoptosis mechanism induced by azadirachtin
Source: Sci Rep. 2017 Oct 16;7:13231. doi: 10.1038/s41598-017-12713-9 (PMC5643380; doi:10.1038/s41598-017-12713-9)
Supplement: Supplementary file 1 — Supplementary File [file 41598_2017_12713_MOESM1_ESM.pdf]

Transcriptome analysis of *Spodoptera frugiperda* Sf9 cells reveals putative apoptosis-related genes and a preliminary apoptosis mechanism induced by azadirachtin

Benshui Shu, Jingjing Zhang, Veeran Sethuraman, Gaofeng Cui, Xin Yi, Guohua Zhong*

Key Laboratory of Crop Integrated Pest Management in South China, Ministry of Agriculture, Key Laboratory of Natural Pesticide and Chemical Biology, Ministry of Education, South China Agricultural University, Guangzhou, People's Republic of China

*Correspondence to: Prof. Guohua Zhong. Key Laboratory of Natural Pesticide and Chemical Biology, Ministry of Education, PR China, South China Agricultural University, Guangzhou 510642, China. E-mail: guohuazhong@scau.edu.cn. Tel: +86-20-85280308; Fax: +86-20-85280203

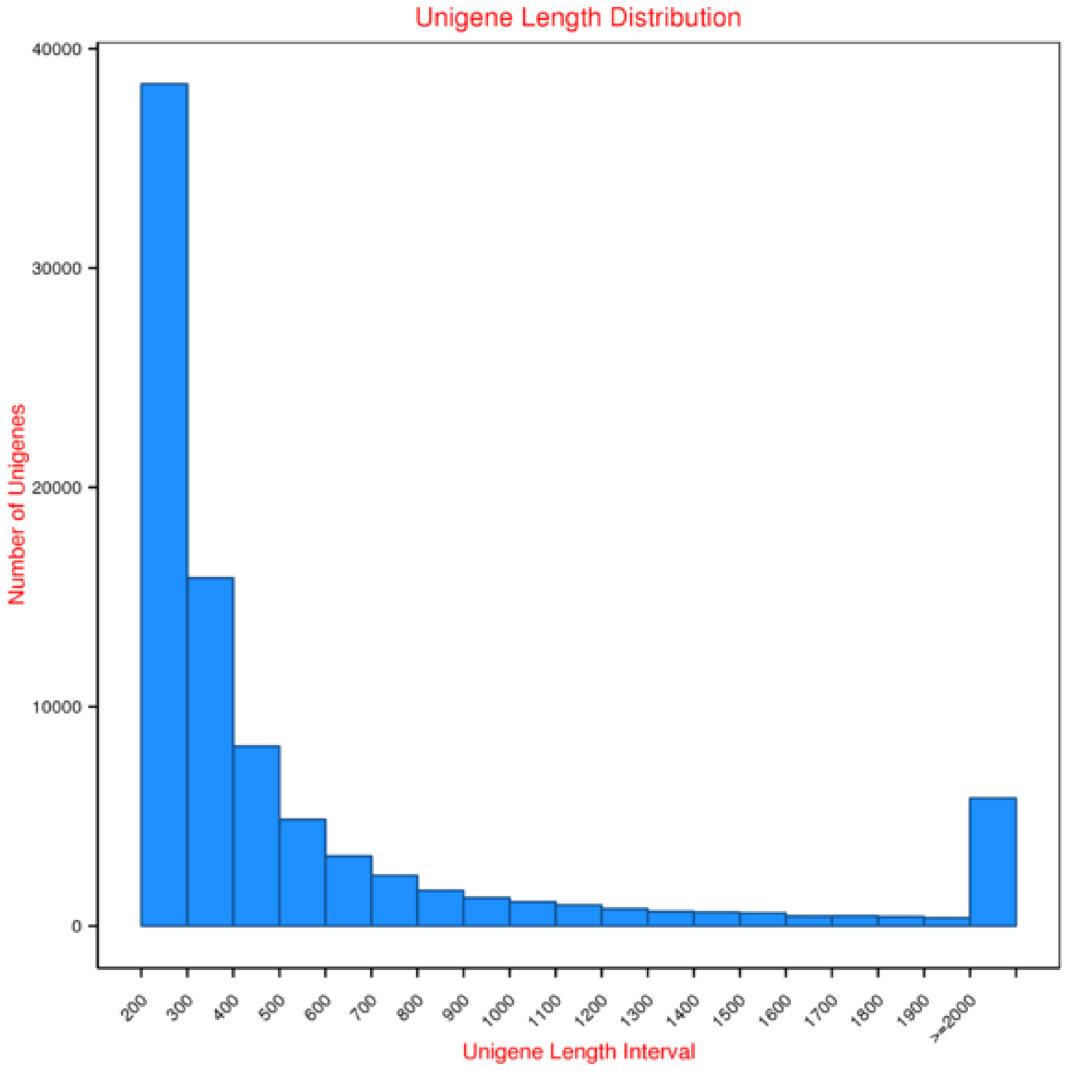

Supplement Figure 1. Histogram presentation of the length distribution of Sf9 cells unigenes. The x-axis indicates unigenes length interval range from 200 nt to >2000 nt. The y-axis indicates the number of unigenes for every length interval.

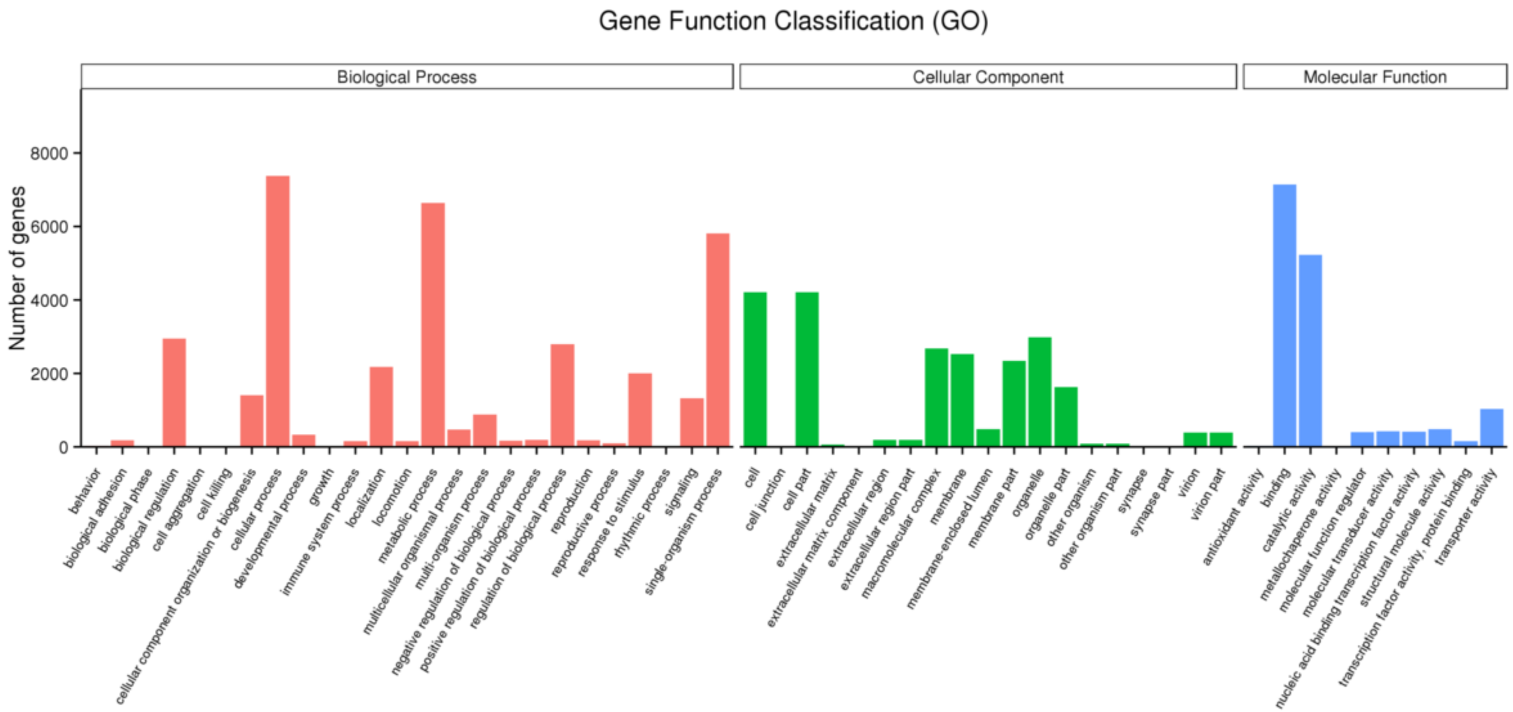

Supplement Figure 2. Gene Ontology (GO) classification of Sf9 cells unigenes. One unigene could be annotated into more than one GO term. Three different classification represent the three basic categories of Go term (from left to right were the biological process, cell composition, molecular functions).

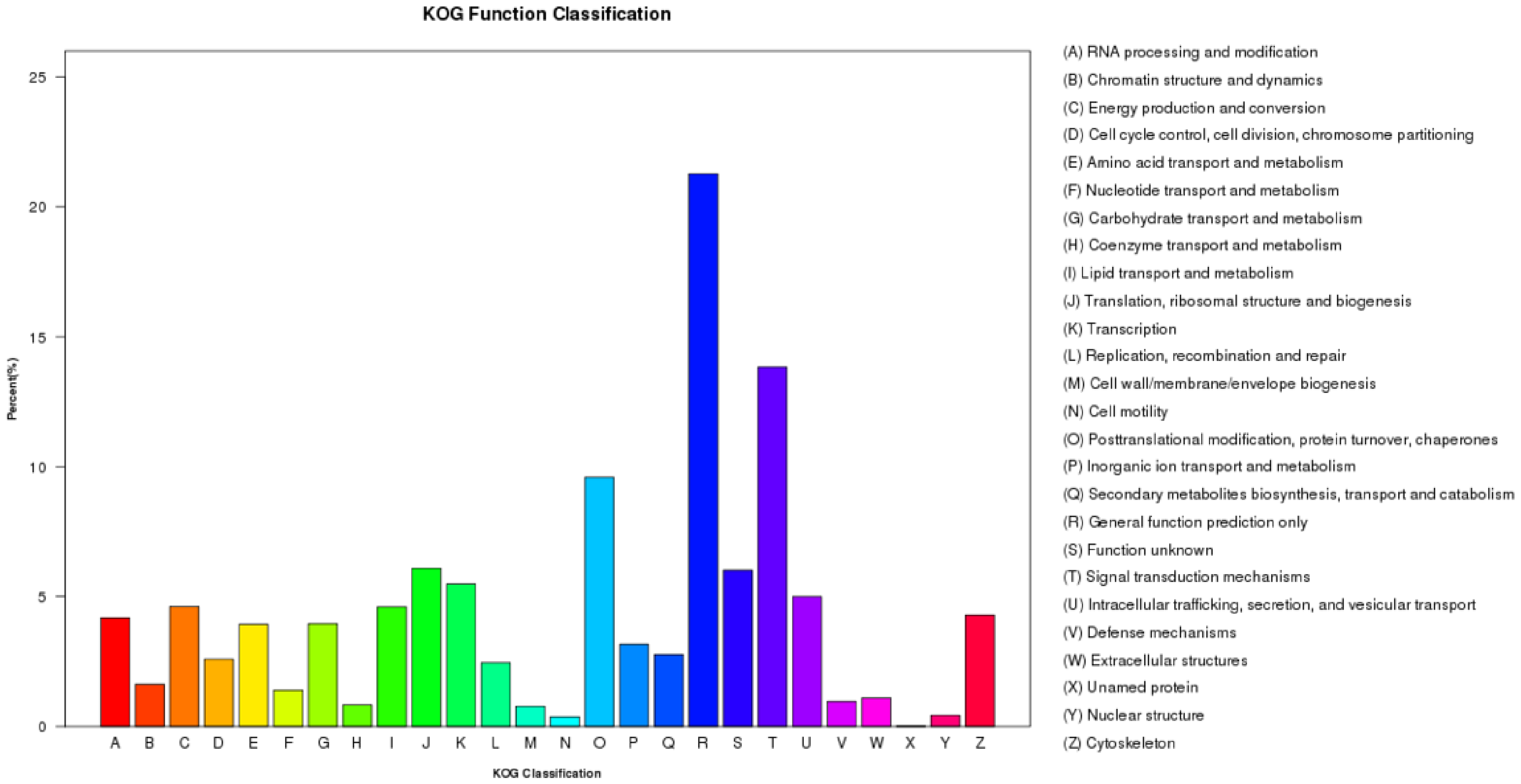

Supplement Figure 3. Histogram presentation of KOG classification of Sf9 cells unigenes. 6031 annotated unigenes were divided into 26 groups. The x-axis indicates the 26 COG categories and the y-axis indicates the ratio for each COG category.

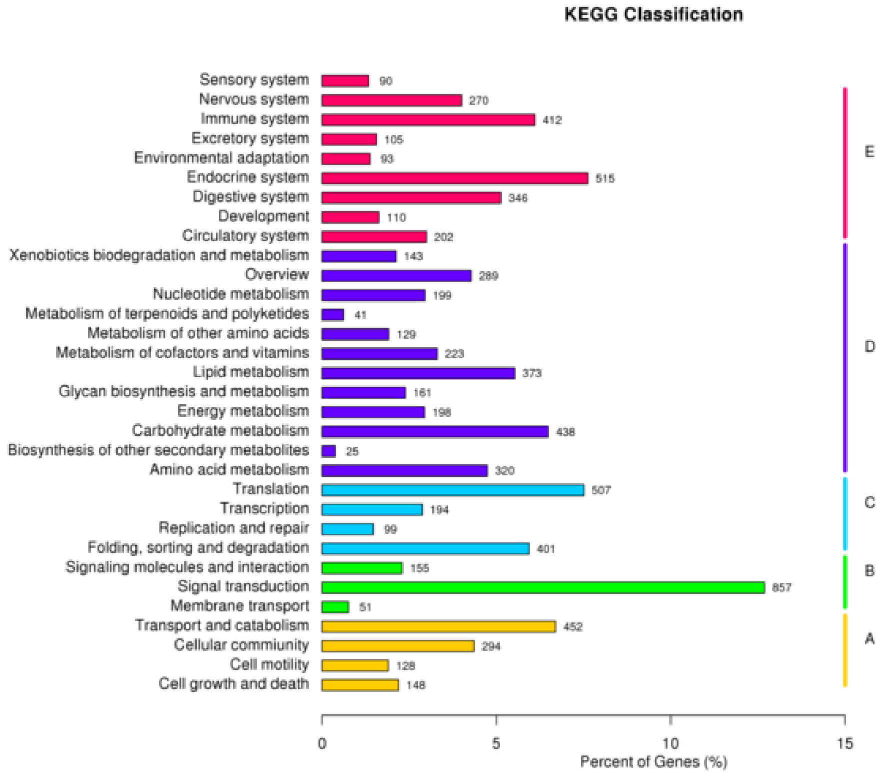

Supplement Figure 4. Kyoto Encyclopedia of Genes and Genomes (KEGG) classification of Sf9 cells unigenes. KEGG metabolic pathways can be divided into five branches: cell process, Environmental Information Processing, the Genetic Information Processing, metabolic, organic system. The y-axis indicates the name of the metabolic pathways and the x-axis indicates the percentage of each metabolic pathways.

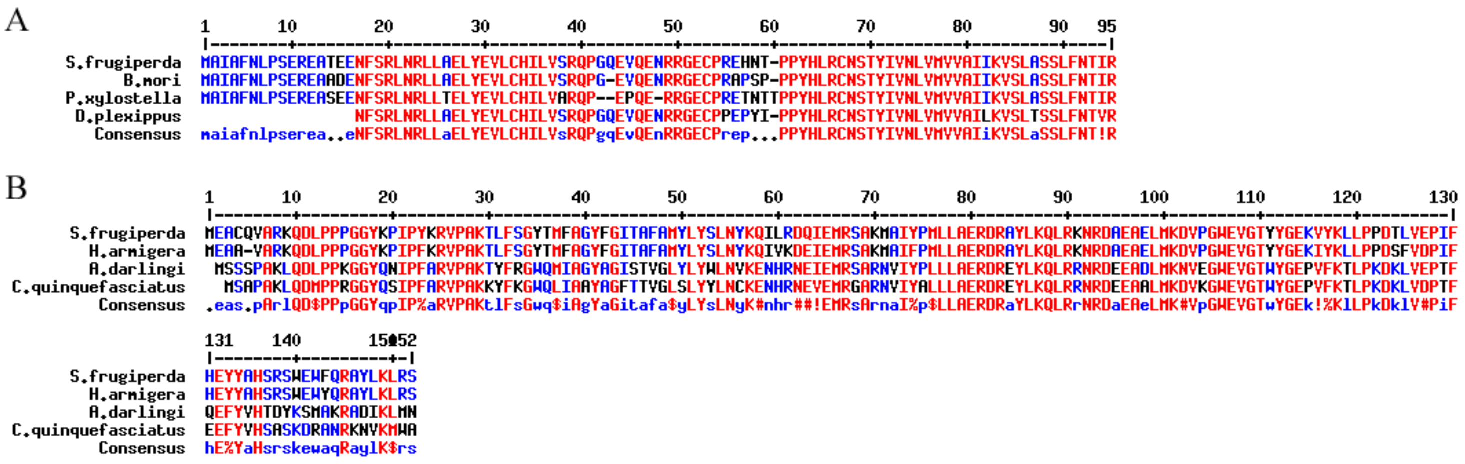

Supplement Figure 5. Multiple sequence alignment of *Sf-IBM1* and *Sf-Grim-19*. The sequences are aligned in http://multalin.toulouse.inra.fr/multalin/multalin.html. Conserved residues in Figure are red, consensus alternatives are blue. A: Amino acid sequences alignment of *Sf-IBM1* and homologous proteins identified in insect. Amino acid sequences from *B.mori Bombyx mori* (NM_001166341.1); *P. xylostella Plutella xylostella* (KF991219.1); D. plexippus *Danaus plexippus* (EHJ77575.1) were aligned. B: The predicted amino acid sequence of *Sf-Grim-19* is shown aligned to *H.armigera Helicoverpa armigera* (ADM64320.1); *C. quinquefasciatus Culex quinquefasciatus* (NP_001037572); *A. darling Anopheles darling* (ETN58336.1).

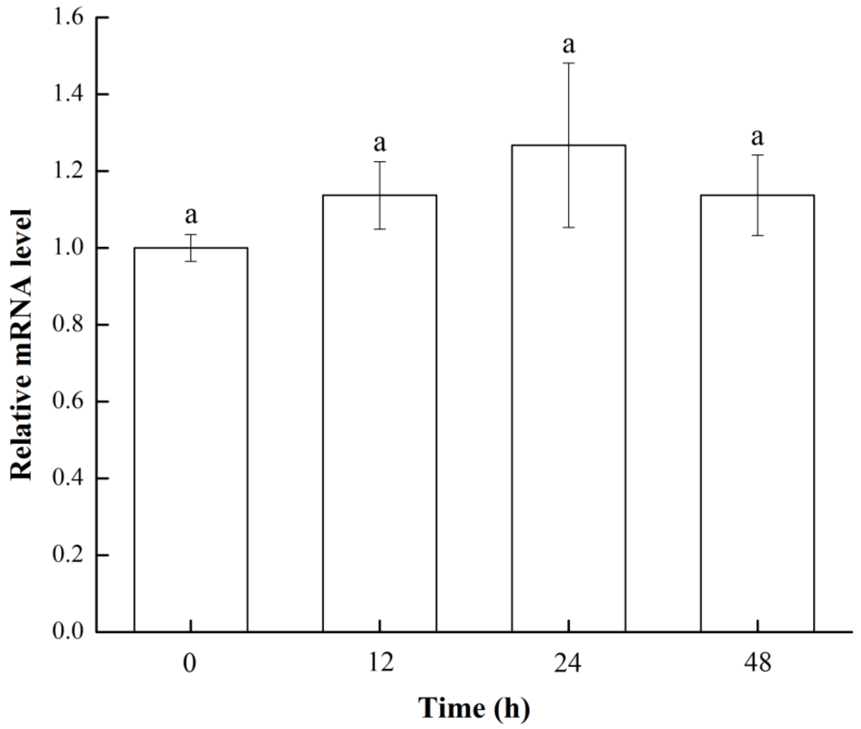

Supplement Figure 6. qRT-PCR analysis of *Sf-Cyt c* between control cells and the cells after treated with azadirachtin at 12 h, 24 h and 48 h. The data are expressed as arithmetic mean ± SEM (n=3). Different letters above bars indicate significant differences between different treatments at the same time ($P<0.05$) by ANOVA followed by DMRT.

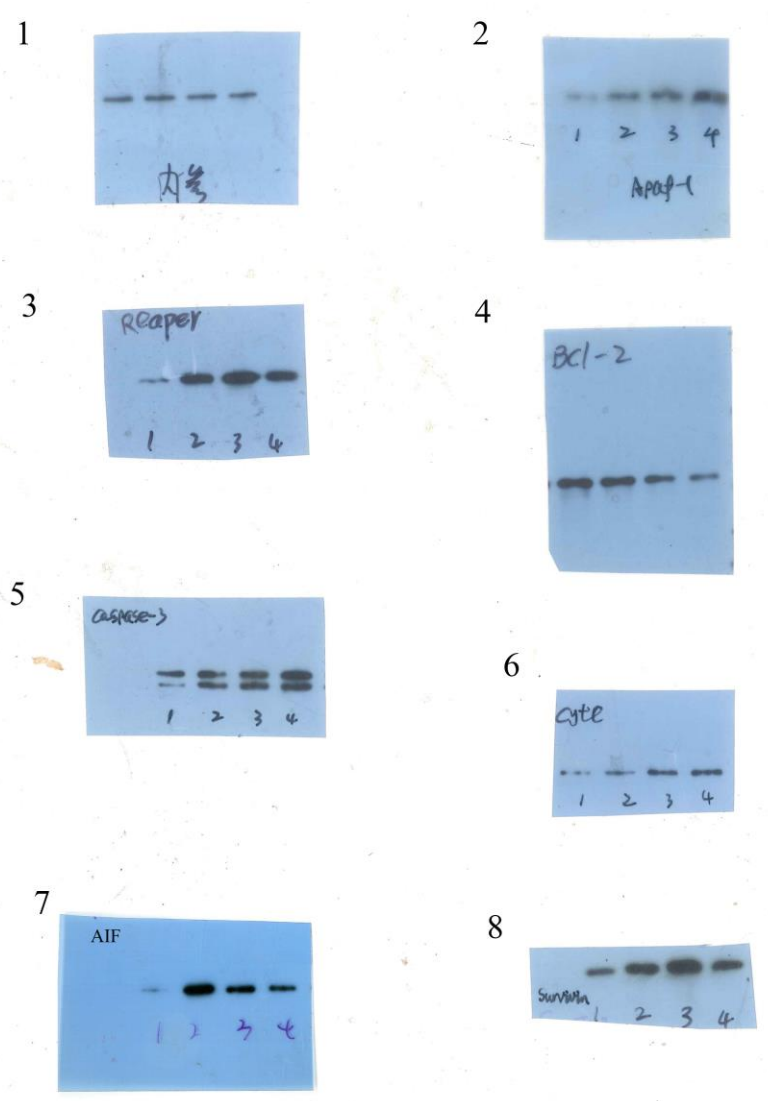

Supplement Figure 7. The raw data of western blot. From 1 to 8 represented GAPGH, Apaf-1, IBM-1, Bcl-2, Caspase-3, Cyt c, AIF and Survivin respectively.

Supplement table 1. Primers used in the paper

Name	**Primers Sequences (5’-3’)**	Name	**Primers Sequences (5’-3’)**
Sf-survivin-F:	GGCATGACAAACAATGTTTTAGA	Sf-Reaper-F	ATGGCTATAGCATTCAACT
Sf-survivin-R:	TCATTTCCTTCCTAGGCATCT	Sf-Reaper-R	TCTGATGGTATTGAAGAGAGA
Sf-IAP-F:	ATGTGGTCGTGTTCCTTACCT	Sf-Bax-inhibitor-F	ATGACTCCCAATCTTCAAACAT
Sf-IAP-R:	TCACGAGAAATATAACCGCAC	Sf-Bax-inhibitor-R	TTCACGTTTGCGGCGGC
Sf-buffy-F:	ATGCGACGGAAACTGAGCA	Sf-caspase-1-F	ATGCTGGACGGAAAACAAGAC
Sf-buffy-R:	TTAGTCGTTGTATGCCGGGTG	Sf-caspase-1-R	TCAGTGGGACTGCTTCTTACC
Sf-Pkar1-F	ATGGAAGAAGAGCAATGTTTAC	Sf-AIF-F	ATGGCTGAGGTTGCGAGTG
Sf-Pkar1-R	TATACAGATAACGAAACGAAGC	Sf-AIF-R	CGTTACCATTTCTTGACGTTCTTA
Sf-Pcdp5-F	ATGGATGACCCAGAATTAGA	Sf-caspase-2-F	ATGACTAATAGCAGCAATAATG
Sf-Pcdp5-R	CTAGAAATCTTCATCGTCTG	Sf-caspase-2-R	TTAGATATTACGCCTGCTTG
Sf-Ras-F	ATGCGTGAATACAAAATAGTC	Sf-Grim-19-F	ATGGAGGCTTGTCAGGTAGC
Sf-Ras-R	TTACAGAATGAGACACTTGAT	Sf-Grim-19-R	AGAGCGCAGCTTGAGGTAA
Sf-Traf 6-F	ATGGATCGAGATAGAAGTAAAGA	Sf-Cyt c-F	TATCCCCGCAGGAAACGC
Sf-Traf 6-R	TCATACACAGCGGATACCCA	Sf-Cyt c-R	GGCGGTTACTTAGTAGCTTCTTT
Sf-Rptor-F	TGACAGAAATGCCTCCTTATC	Sf-cytochrome c-RT-F	GCTTCTCATACTCAGATGCCAACA
Sf-Rptor-R	TCAGTTGCGCCTGTCCAA	Sf-cytochrome c-RT-R	CTTCTTTTAGGTAGGCGATGAGGT
Sf-Reaper-RT-F	TCCAGGAGAATAGGCGAGGTG	Sf-AIF1-RT-F	CAAGCACTACACGCACCAGAG
Sf-Reaper-RT-R	CGAGGGAGACCTTTATGATGGC	Sf-AIF1-RT-R	CTGCCGAGAAGACACCCACT
Sf-caspase-2-RT-F	TAGCAGCAATAATGGAGGACGC	Sf-caspase-5-RT-F	GATACTGGGACTTGGTGCGTGAT
Sf-caspase-2-RT-R	CTCGGTACTTGTGGTTGGTGTTG	Sf-caspase-5-RT-R	TGCGTGTTGTTTCTGTTGGGTT
Sf-IAP-RT-F	AAAACCGACAACCACGACACC	Sf-Buffy-RT-F	TGGCGAAGAAGATGACGAGTTT
Sf-IAP-RT-R	CCCTCCACCCACCTCATAATCT	Sf-Buffy-RT-R	CGCTGCTTGAGACCATAGTGC

Sf-Survivin-RT-F	TTGGGCGGAGCACAAAAGC	Sf-Apaf1-RT-F	ACCGAACGAGATTATGGAGCAT
Sf-Survivin-RT-R	GCACTGCCTTTGCCTTCTCATC	Sf-Apaf1-RT-R	AACGACCCGACTATCATCCGT
Sf-GAPDH-RT-F	TTGACGGACCCTCTGGAAAA	Sf-GAPDH-RT-R	ACGTTAGCAACGGGAACACG

Supplement table 2: Statistics of the Sf9 cells transcriptome assembly compared to Sf_TR2012b

	Transcripts of Sf9 transcriptome	Unigenes of Sf9 transcriptome	Sf_GATC _Clusters	Sf_TR 2012b
Sequence numbers	103977	87860	183373	54976
N50 (nt)	2066	1182	408	876
N90 (nt)	288	258	295	400
Total Nucleotides (nt)	90689140	59027951	61002208	36925829

nt: nucleotide
